# Pyuric diabetic patients: A tertiary centre experience from Karachi

**DOI:** 10.12669/pjms.301.4356

**Published:** 2014

**Authors:** Huma Mamun Mahmud, Sobia Qureshi, Darshan Kumar, Syed Farman

**Affiliations:** 1 Dr. Huma Mamun Mahmud, MBBS, MCPS (Medicine), FCPS (Nephrology), Assistant Professor, Consultant Nephrologist, Department of Medicine, Dow International Medical College, Dow University of Heath Sciences, Dow International Medical College (Ojha Campus), DUHS, Suparco Road, off Main University Road, Gulzar-e-Hijri, Scheme 33, Karachi, Pakistan.; 2 Dr. Sobia Qureshi, MBBS, Senior Medical Officer, Dow International Medical College, Dow University of Heath Sciences, Dow International Medical College (Ojha Campus), DUHS, Suparco Road, off Main University Road, Gulzar-e-Hijri, Scheme 33, Karachi, Pakistan.; 3 Dr. Darshan Kumar, MBBS, FCPS (Medicine), Assistant Professor, National Institute of Diabetes and Endocrinology. Dow International Medical College, Dow University of Heath Sciences, Dow International Medical College (Ojha Campus), DUHS, Suparco Road, off Main University Road, Gulzar-e-Hijri, Scheme 33, Karachi, Pakistan.; 4 Dr. Syed Farman, Dow International Medical College, Dow University of Heath Sciences, Dow International Medical College (Ojha Campus), DUHS, Suparco Road, off Main University Road, Gulzar-e-Hijri, Scheme 33, Karachi, Pakistan.

**Keywords:** Diabetes, Sterile pyuria, UTI

## Abstract

***Objectives: ***(1) To determine frequency of urinary tract infection among pyuric diabetic patients. (2) To determine sterile pyuria frequency among pyuric diabetic patients. (3) To determine factors predisposing to urinary tract infection.

***Methods: ***This is a non randomized, prospective observational study done in tertiary care set up of Dow University of Health Sciences, Karachi. Data collection done from June 2013 till August 2013. Sampling was done by convenient method, sample size of 97. Inclusion criteria was all adult (above 16) patients with diabetes mellitus and pyuria (more than 4 pus cells /HPF) whose urine culture report was also available. Verbal consent was sought from patients. All data was collected on a Performa. Data was maintained and analyzed on SPSS version 16.

***Results: ***Total number of pyuric diabetic patients in study was 97. Frequency of Urinary tract infection was 59/97 (60.82%), prevalence of culture negative sterile pyuria was found 38/97 (39.17%). Urinary tract infection was found to be more in females with lower urinary tract symptoms and flank pains. Stone disease, obstructed pelvicalyceal system, proteinuria, high serum creatinine and positive nitrites were found more in culture positive patients than in culture negative pyuric patients.

***Conclusions:*** Pyuric diabetic patients in our study population were found to have culture positive UTI in 60.82% and culture negative sterile pyuria among 39.17% of patients. UTI was found more in females, in symptomatic patient and with abnormal urinary tract anatomy and function.

## INTRODUCTION

Urine detailed report (urine D/R) is an important and early investigation for evaluation and screening of a number of diseases. Urine D/R is also an initial and important assessment step in patients having lower urinary tract symptoms (LUTS) or unexplained febrile illnesses. Presence of pus cells in urine defined as pyuria is an important accompaniment of bacteriuria which may be asymptomatic or can indicate toward underlying urinary tract infection (UTI). Pyuria is significant if there are more than 4 pus cells/HPF in a centrifuged urine sample. Presence of pyuria in presence of lower urinary tract symptoms and positive nitrite accompanied by bacteria is highly suggestive of urinary tract infection. Sterile pyuria is a known entity which indicates presence of pus cells in urine without an evidence of infection on routine standard culture.

A number of infections which are not picked up by routine urine culture are found to be associated with sterile pyuria like genitourinary tuberculosis^1^^,^^2^, infection with fastidious growing organisms like Chlamydia, Urea plasma urealyticum^3^, Known systemic diseases associated with sterile pyuria are SLE^4^, Kawasaki’s disease^5^, Sickle cell disease^6^ and Reiters syndrome.^7^

Few structural abnormalities of kidneys well known to be associated with sterile pyuria are stone disease,^8^ incontinence, indwelling catheterization, renal papillary necrosis, poly cystic kidneys, renal neoplasms, post treatment with antibiotics for 2 weeks or inadequately treated UTI and sometimes tubulointerstitial nephritis.^9^

Prevalence of UTI among diabetics is reported to be 25.3% in a recently published article from Saudi Arabia,^10^ another study from Italy has mentioned about asymptomatic bacteriuria among diabetics.^11^

At present prevalence of patients with sterile pyuria among diabetics, association of diabetes with pyuria, whether or not pyuria need antibiotics, are not well described in literature.. Currently no definite guidelines exist how to evaluate patients with pyuria or sterile pyuria.

This study aim was to help in estimating the problem load among diabetics and in identification of patients at risk of urinary tract infection and is supposed to be used as reference in designing protocols and guidelines in evaluation and management of diabetic patients.

## METHODS

This is a convenience sample based observational study done in tertiary care set up of Dow University of Health Sciences, Karachi. Data collection was done from June 2013 till August 2013. 

All adult (above 16) patients with diabetes mellitus and pyuria (more than 4 pus cells /HPF) whose urine culture was also available, were included in study. Urine D/R and C/S being sent at same time. Patients included were selected from both outpatient and inpatient departments. History of patient’s age, diabetes duration, lower urinary tract symptoms, fever, flank pain and known comorbids, stone disease, benign prostatic hypertrophy was collected through direct interview and filled on a proforma. Laboratory information was gathered from file regarding hemoglobin A1C, urine D/R, Urine C/S, serum Creatinine, proteinuria, glucosuria, Ultrasound Kidneys, ureter and bladder.

**Table-I:** Demographic data of study population

	*Total no of patients*	*Pyuric diabetic patients with UTI*	*Diabetic pyuric patients with sterile pyuria*
No of patients	97	59(60.8%)	38 (39.1%)
Mean age	50.97+19.34	55.97 SD+ 11.30	55.53 SD +9.88
M:F, gender	1:1.93	1:2.27	1:1.37
Patients seen in OPD	79	46	33
Patients seen in ward	18	13	05
Diabetes duration in years	7.15 SD + 6.26	6.85 SD+6.03 Missing value-13	7.46 SD+ 6.50 Missing value -08

**Table-II:** Clinical parameters of diabetic pyuric patients

	*Pyuric diabetic patients with UTI*	*Sterile pyuria in diabetic pyuric patients*
Total no	59	38
BPH among males	4/18(22.2%)	01/16(2.6%)
Past history of UTI	36/59 (61%)	20/38 (52.6%)
LUTS	39/59 (66.1%)	17/38 (44.7%)
Fever	30/59 (50.84%)	11/38 (28.9%)
Flank pain	17/59 (28.81%)	09/38 (23.68%)

**Table-III:** Laboratory results on study population

	*Pyuric diabetic patients with UTI*	*Sterile pyuria in diabetic pyuric patients*
Total no of patients	59	38
HbA1C	6.1 SD + 4.66	7.1 SD+ 3.75
Glucosuria	17/59(28.8%)	14/38(36.8%)
Proteinuria on dipstick	41/59(69.5%)	19/38(50%)
Nitrite positive on dipstick	08/59(13.6%)	01/38(2.6%)
Obstructed pelvicalyceal system on ultrasound	08/59(13.6%)	01/38(2.6%)
Serum creatinine	Mean 1.73 SD+ 1.24	Mean 1.56 SD+ 2.2

Fresh urine sample was examined in lab on duri Urine analyzer, centrifugation done at 2500 rpm for 5 minutes. Microscopy done at high power field (40X100) for cell counts. Mid stream specimen of urine was cultured on cystein, Lactose, dextrose deficient agar for 24 hours. Sample size collected was 97. Verbal consent was sought from patients before interviewing and patients were given information sheet. All data was collected on a proforma. Data was maintained and analyzed on SpSS version 16.

## RESULTS

Number of patients approached was 123 how ever 26 were those on whom data was incomplete so number of patients finally included in study was 97. Demographics given in Table I

Mean Hba1c in our patients was 6.61+ 4.205. Out of 33 male patients benign prostatic hypertrophy was known in 05(15%) patients. Past history of culture positive urinary tract infection was seen in 56. Fever was present as a symptom in 41/97 (42.2%).

Flank pain in 26/97 (26.8%). Lower urinary tract symptoms defined as either frequency, urgency, dysuria or hesitancy were present in 56/97 (57.7%) 

UTI was found in 59/97 (60.8%) pyuric patients. In this group of pyuric diabetic patients with UTI mean age was 55.97+ 11.3, M: F was 1: 2.27, mean diabetes duration was 6.85 SD+ 6.03, Benign prostatic hypertrophy was found in 4/18 (22.2%), past history of UTI was in 36/59 (61%), Lower urinary tract symptoms in 39/59(66.1%), fever in 30/59 (50.84%) and flank pain in 17/59 (28.8%). On lab evaluation mean HbA1c was 6.1 SD + 4.66, glucosuria was found in 17/59 (28.8%), proteinuria in 41/59(69.5%), nitrite positivity in 8/59 (13.6%), obstructed tract on ultrasound was seen in 8/59 (13.6%) and mean serum creatinine was found 1.73SD + 1.24.

Sterile pyuria was seen in 38/97(39.1%). In this group of pyuric diabetic patients with no growth on standard urine cultures benign prostatic hypertrophy was seen in 01/16(2.6%), past history of urinary tract infection was found in 20/38(52.6%), lower urinary tract symptoms in 17/38 (44.7%), fever in 11/38(28.9%), pain in 9/38(23.6%).

On lab evaluation HbA1C was 7.1SD+3.75, glucosuria was found in 14/38(36.8%), proteinuria in19/38(50%), nitrite positivity in 1/38(2.6%), obstructed urinary tract was found on ultrasound in 1/38(2.6%) and mean serum creatinine was 1.56SD +2.2.

These results shows that UTI was more common in females and in symptomatic patients. Proteinuria, higher serum creatinine, nitrite positivity and obstructed urinary tract were also seen more in patients with UTI.

## DISCUSSION

 Overall reported frequency of UTI among diabetics is 25.3% while 41.1% among these were females.^10^ In present study we have observed frequency of UTI in more than half of diabetic pyuric patients. This could be explained on basis of our selection criteria, our patients were diabetic and all were pyuric on urine D/R which itself is a marker of inflammation. We were unable to find any published study which describe frequency of UTI among pyuric diabetic patients. 

Available evidences does not support antimicrobial treatment of asymptomatic bacteriuria among diabetics.^12^ Likelihood of UTI increases if diabetic patient with pyuria is symptomatic.^13^ Early detection and treatment of symptomatic UTI in diabetics is required to prevent pyelonephritis and renal abscesses.^14^

Our observations of UTI in patients with enlarged prostate, or past history of UTI was found consistent with findings observed by others, that is UTI suggestive in diabetics is more likely to consisyent with actual UTI when they are symptomatic, and same is true for patients with obstruction as with BPH.^9^^,^^13^ We have also observed proteinturia in about 70% of our UTI patients.

Females are known to be more at risk of UTI,^15^ this was redescribed with our present study with M:F ratio of 1: 2.27 However, gender discrimination was found not that important in patients with sterile pyuria, ratio being 1: 1.37. Out of our 97 diabetic pyuric patients 38 (39.1%) were without any growth on standard urine culture or said to have sterile pyuria. Sterile pyuria is not well studied through researches in literature. A number of diseases have been associated with sterile pyuria as defined in introduction.^1^^-^^6^^.^

Among these 38 diabetic pyuric patients with no growth we have found only one patient who had an obstructed pelvicalyceal system on ultrasound which is also known to be associated with sterile pyuria.^8^^,^^9^ Other diseases known to be associated with sterile pyuria like SLE, Reiters syndrome, Kawasaki’s disease, indwelling catheter, renal papillary necrosis, stone disease, none of these were found in our patients as per history and through case sheet evaluation.

This raises the question that are diabetics themselves having a higher prevalence of sterile pyuria than general population, answer of which cannot be found through this study and it need a more controlled randomized and comparative study. One study from Italy has mentioned about asymptomatic bacteriuria among diabetics.^11^ Recent retrospective study on genitourinary tuberculosis done locally in Pakistan has defined prevalence of sterile pyuria in genitourinary tuberculosis to be 19%.^16^

Through our study on selected pyuric patients we have tried to show that among all of diabetic pyuric patients only 60.8% of our patients were with a positive culture report and rest were with no growth on standard urine cultures. Therefore, it is reasonable to suggest that diabetic pyuric patients need treatment in females, especially when symptomatic or with abnormal anatomy and function of urinary tract.

## CONCLUSION

Pyuric diabetic patients in our study population were found to have culture positive UTI in 60.82% and culture negative sterile pyuria among 39.17% of patients. UTI was found more in females, in symptomatic patient and with abnormal urinary tract anatomy and function. Further studies are needed to define if diabetes itself is a cause for sterile pyuria. 

## Authors Contribution:

Dr. Huma Mamun Mahmud: Topic selection, Data collection, Defining materials and methods, Writing discussion, Contribution in final paper writing.

Dr. Sobia Qureshi: Data collection, Data maintenance on SPSS and Result writing.

Dr. Darshan Kumar: Data collection, Writing introduction, Literature search.

Dr. Syed Farman: Data collection, Literature search, Data entry on SPSS and Writing abstract.

## Notes

***Limitations of study: ***Our study is having small number of patients and sampling is done for convenience and this may be a reason to have bias.

## Notes

***Source of funding:*** Primary author for paper publication. For research-none.
